# Plant embryogenesis in medieval thought: Dino del Garbo’s commentary on Hippocrates’ *De natura foetus*

**DOI:** 10.1007/s40656-026-00747-0

**Published:** 2026-09-28

**Authors:** Marilena Panarelli

**Affiliations:** Università di Palermo, Palermo, Italy

**Keywords:** Medieval plant science, Plant embryogenesis, Scholastic medicine, Dino del Garbo, Hippocratic embriology

## Abstract

This paper investigates the *Recollectiones in Hippocratem De natura foetus*, a unique Latin commentary on the Hippocratic embryological treatise, composed in the early fourteenth century by the Florentine physician Dino del Garbo. While the original Hippocratic text is concise, Dino expands it significantly, offering a rich and systematic exploration of the analogies between plant and animal generation. His commentary stands out not only as the sole known Latin interpretation of this Hippocratic work, but also as an exceptional instance of botanical inquiry within the medical-philosophical framework connected to the Bolognese medical school. Focusing on passages that address plant embryonic development, the study highlights Dino’s systematic use of analogies between animal and vegetal reproduction, particularly through his discussion of seed-based generation and vegetative propagation. Philosophical works such as the Pseudo-Aristotelian *De plantis* and, though not explicitly cited, Albert the Great’s *De vegetabilibus* appear to inform the broader conceptual background of the commentary. His reflections on seed anatomy, sexual differentiation, and the distribution of the vegetative soul reveal an effort to reconcile observations in plant science with a rigorous philosophical approach. By integrating Hippocratic empiricism with Aristotelian natural philosophy, Dino del Garbo offers a rare contribution to medieval plant science—one that bridges medical theory, botanical physiology, and metaphysical inquiry.

## Introduction

The short treatise attributed to Hippocrates and known by various titles such as *De natura foetus*, *De natura embryonis*, and *De natura puerorum* is entirely devoted to embryological matters, and it notably employs analogies with the plant world to explain human generation (Giorgianni, 2006).^1^ The Chaps. 22–27 of this treatise are in fact entirely devoted to botanical inquiry (Lonie, 1969) adopting a reasoning by analogy, comparing embryogenesis in plants and animals. This use of analogy is characteristic of the Hippocratic method, which, in the absence of direct access to the object of inquiry, resorts to the examination of analogous phenomena, with the assumption that the results may be transferred back to the original domain of investigation (Giorgianni 2012a, pp. 14–17). As outlined by Pearl Kibre (1980); Giorgianni (2012b), who have extensively studied the transmission and reception of the Latin translations of Hippocrates’ works, the text was translated directly from Greek into Latin in the thirteenth century by Bartholomew of Messina, at the court and by the commission of King Manfred of Sicily.^2^ This evidence is provided by the introductory words to Bartolomew’s translation in certain manuscripts, such as the *Clm 39*, whose *incipit* explicitly confirm this attribution.^3^

The *Recollectiones in Hippocratem De natura foetus* (Dino del Garbo, 1518) is the only known Latin commentary on this Hippocratic work, and it was compiled by the Florentine physician Dino del Garbo in 1310, more precisely on 10 October 1310, as is attested by Giuliano de Preunti, the reporter of the work.^4^ Dino del Garbo (?—ca. 1327) was educated under Taddeo Alderotti in both the liberal arts—thus philosophy—and medicine, whose distinguished career included teaching appointments at Bologna, Siena, and Padua (Siraisi, 1981; Chandelier, 2017). However, the existence of this commentary is interesting not merely because it seems to be the only one in Latin, but also because it contains the only botanical enquiry among the medical and philosophical works related to the Bologna medical school^5^.

In contrast to the brevity of Hippocrates original work, Dino’s commentary expands significantly on the material, offering a richer and more detailed interpretation, particularly with regard to the analogies between animal and vegetal generation.^6^ It focuses on the latter, making the analogy between the two a systematic instrument of inquiry. As noted by Nancy Siraisi (1981, p. 63), Dino del Garbo incorporates a substantial number of references to philosophical sources, giving the text a rigorous theoretical structure.

This article will examine the section of Dino del Garbo’s *Recollectiones* that addresses topics related to what is nowadays understood as plant embryonic development, paying particular attention to the various vegetative stages of plant growth.

The section opens with a preliminary consideration that serves as the foundation for the entire argument:

Plants have two modes of reproduction: some grow from seeds sown in the soil, while others reproduce through explantation.^7^

This fundamental distinction enables a dual approach to the question of embryonic development in plants: one related to seed reproduction and the other to vegetative propagation by cutting (*per explantationem*).

By analysing these passages, the present paper seeks to assess the contribution of Dino’s commentary to the development of medieval plant science, situating it within the broader intellectual context of medieval botanical studies. Among the various sources considered, particular attention is paid to the pseudo-Aristotelian *De plantis*, which is considered the foundational text of this line of tradition, and to Albert the Great’s *De vegetabilibus*, which made the most significant contribution to the development of medieval plant science.^8^

## Reproduction by seeds: the three stages of embryonic development

Regarding seed-based generation, the analysis explains the embryological development of plants in comparison to that of animals.^9^ The analogy between plant and animal generation, as outlined by Dino del Garbo, unfolds on two distinct levels: location (*quantum ad locum*) and developmental stages (*quantum ad dispositiones*). Regarding the first point, just as animals develop in a specific location—the womb (*animalia habent proprium locum in matrice*)—so too do plants originate from seeds in the soil (*vegetabilia in terra*). This parallel resembles the traditional metaphor of the earth as the ‘mother’ of plants, insofar as they receive nourishment and growth from it, mirroring the role of the womb in animal gestation.^10^

Following Hippocrates, Dino lists three different kinds of analogous developmental stages.

The first concerns the initial physical changes that the seed and the semen undergo.^11^ In both cases, the generative process begins with swelling and expansion (*inflatur et tumescit*), which are attributed to the action of heat on moisture. In seeds, this transformation is explained by the interaction between a minimal innate heat and the moisture drawn from the surrounding soil, which is further intensified by the warmth attributed to celestial bodies. Similarly, in animals, the semen initially lacks sufficient generative force. It then undergoes a comparable process of activation through the mixture of male and female reproductive material and the subsequent thickening and heating within the womb. Following Hippocrates, Dino notes that generation begins when the semen becomes thickened and heated, resulting in a swelling analogous to that observed in seeds.

The second developmental stage concerns the dynamic role of moisture in the development of the embryo. Just as the generative power in the seed draws moisture from the surrounding soil, so does the semen attract moisture from the maternal environment when deposited in the womb. The moisture involved in plant generation is described as twofold: one part is intrinsic to the seed itself, while the other is extrinsically absorbed from the soil. According to Dino’s interpretation of Hippocrates, it is precisely this dual contribution that enables the seed to develop: the internal moisture itself is insufficient and must be supplemented and intensified by that drawn from the earth: the inherent generative power—initially weak within the seed—is strengthened and amplified through the attraction of external moisture. This model is then extended to animal reproduction, which is described as operating in exactly the same way: the semen in the womb, like the seed in the soil, relies on both its own inherent potential and the additional support of its surrounding to initiate embryogenesis.

The third developmental stage identified in the seed occurs when—after swelling and being invigorated by its internal generative power—the seed bursts open (*erumpitur*), initiating the emergence of the first structures. Dino draws a parallel with animal reproduction: just as the seed bursts open once its inner force has matured, so too does the semen, once swollen, break open, creating a path for development. From this burst, a structure emerges that is functionally analogous to the plant’s first leaves: a thin membrane forms around the seed, protecting it from external corruption, just as the initial leaves safeguard the plant. This stage illustrates a key functional analogy between the early protective mechanisms in plant and animal development.

The role of these early leaf-like structures as protective agents highlights a functional correspondence between vegetal and animal development. As will be examined later, this aspect is particularly emphasized by Hippocrates, and Dino develops an extended argument on the protective and functional role of seed leaves in the broader framework of plant embryology.

At this point, however, the commentary turns to a brief digression concerning the question of sexual differentiation in plant seeds.

### The distinction between male and female principles in seeds

Dino refers to a significant difference: unlike in animals, in which male and female generative principles are distinctly separated, these principles are not individuated but rather coexist in a confused or blended form within the same seed in plants:

And these three stages (*dispositiones*) are set out by Hippocrates in the text, as is clear. However, although plants and animals share these stages, there is a fundamental difference between them: in seeds and plants, the generative power of the male is not distinct from that of the female, as it is in animals, but rather the two are blended in the same seed. Nevertheless, even though these powers are thus mixed in the seed, it is still possible to distinguish one part that possesses more of the active principle, and another that plays the role of the passive principle. In this way, the active principle is stronger in one part—which can analogically be referred to as “male”– while in another part the passive principle predominates, and this is similar to the “female”.^12^

Interestingly, despite this blending, Dino argues that even in a single plant seed, there remains a certain functional difference: one part of the seed may predominantly exhibit an active (male-like) generative power, while another part may correspond to a passive (female-like) capacity. This internal distinction within a unified structure allows him to speak of a quasi-sexual polarity even in the absence of distinct sexual organs. He supports this idea with the example of a wheat grain, noting that the active principle is concentrated in the tip (*cuspis*) of the grain. This argument is reinforced by the empirical observation of the behavior of ants, which are said to gnaw off the grain’s tip when storing it underground to prevent germination, thus effectively removing the part that holds the active generative force.

However, a comparable line of argument can already be found in Albert the Great,^13^ who, in his *De vegetabilibus*, articulates a similar view on the internal differentiation of generative powers in the seed. He states that it is possible to retrace a kind of imitative (*non vera sed longe imitativa*) sexual virtue in it^14^, recognizing that the male principle can be found in the hotter part of the seed while the female principle is found in the colder part. These two texts may fruitfully be compared, as they both use a remarkably similar conceptual framework to describe the internal dynamics of plant reproduction. While Dino del Garbo does not explicitly cite Albert the Great, the structural and terminological similarities between their accounts suggests that Dino has been directly or indirectly influenced by Albert’s *De vegetabilibus*.

However, Dino del Garbo’s position appears more defined—and, in a certain sense, more assertive—than that of Albert the Great. While Albert acknowledges a resemblance to male and female principles in seeds, he does so explicitly in metaphorical terms. By contrast, Dino attributes a concrete existence to this internal polarity. He not only distinguishes between the active and passive principles in the seed but also identifies specific regions (such as the tip of the wheat grain) where these principles are more or less concentrated, suggesting a more physically grounded interpretation.

It is precisely on the basis of this internal differentiation in the seed that the subsequent formation of the various parts of the plant is explained. The active and passive principles serve not merely as metaphors for generative capacity, but as the foundation for understanding the plant’s embryological organization. This conceptual framework informs the account of how specific vegetal structures emerge during development—an aspect that will be elaborated on in the following section.

### The formation of plant parts: between what appears and what truly is

To explain the order in which the various parts of the plant come into being, Dino del Garbo invokes the conceptual distinction between generation according to actual formation (*generatio secundum veritatem*) and generation according to perceptible manifestation (*generatio secundum apparentiam*). The latter is defined as imperfect generation (*imperfecta*), while the former is defined as perfect generation (*perfecta*). This distinction enables him to address the discrepancy between the internal, essential progression of development and the external order in which the parts first become visible:

From this, it becomes clear that the generation of the stem is prior, both absolutely and by nature, to that of the leaf and the root, and that it is completed earlier. Although the leaf appears before the stem, it is not perfected first, but later. Similarly, although the root appears later than the leaf, it is in fact completed earlier, because those initial leaves are not truly perfected and fall. True leaves are regenerated later, emerging from a bud that subsequently produces genuine foliage. Thus, what Hippocrates states here is valid only regarding imperfect generation and generation according to appearance, and it concerns leaves that are not yet true leaves.^15^

Dino del Garbo refines the Hippocratic assertion that the first parts that appear during plant development are the leaves by distinguishing between what appears first and what is in fact generated first by nature. He concedes that a leaf-like structure is the earliest visible formation, yet he insists that this is not a true leaf, but a fleeting form resulting from the lighter, more volatile moisture concentrated in the apical region of the seed. In doing so, he somehow seems to anticipate the later botanical differentiation between true leaves and cotyledons. Cotyledons are differentiated from true leaves and are defined as embryo or seed leaf which “may themselves be a source of nutritional reserves or may aid the embryo in metabolizing nutrition stored elsewhere in the seed” (Encyclopaedia Britannica, 2021). The now widely recognized metabolic function of cotyledons resonates with Dino’s interpretation of the seed leaf as the first outcome of the digestion of the excess watery moisture present in the seed:

And the reason for this is that the part referred to as the leaf—though not a true one—is generated from the more subtle, watery, and airy moisture, whereas the root is generated from the denser, more earthy and terrestrial portion of the moisture, which descends downward. Now, since the seed initially contains a great amount of aqueous and airy moisture, this moisture, seeking an outlet when the seed becomes swollen—its own moisture having been increased by additional moisture drawn from the earth—and due to the ebullition caused by the seed’s natural heat, it is the more subtle, watery, and airy part that is more inclined to break forth from the seed; hence, the generation of this kind of leaf appears first.^16^

The apical region, previously identified by Dino as the seat of the more active, “masculine” principle, is also where the *calidum naturale*—the seed’s innate heat– exerts its initial influence. As this natural heat causes the aerial and aqueous moisture to swell and seek release, it results in the early emergence of the foliar structure. The more substantial vegetative parts, such as the stem and root, originate from denser, terrestrial components and follow in the actual sequence of development.

His account thus allows him to assert the primacy of the stem (*stipes*) and of the root (*radix*) in terms of true generative order (*generatio secundum veritatem*), and to describe them as perfectly generated. At the same time, he interprets the Hippocratic statement regarding the priority of the leaves as referring to the imperfect generation of seed leaves, which are merely a substitute for the true leaves. Despite the seed leaves, which do not correspond to the true leaves, the stem and the root coincide with the fundamental structure that will be present also in the perfected plant. By once again resorting to the comparison with animals, true leaves are likened to animal hairs (*pili*), which are the part formed last, serving as a defense mechanism.

### From seed to seed: the interaction of moisture and heat in the generation of plant parts

Plant generation is understood as resulting from the interaction of heat with moisture in the philosophical tradition concerned with vegetal realm. This concept, already present in this Hippocratic work, finds a more systematic formulation in Aristotle’s *Meteorologica* (Book IV) and in the pseudo-Aristotelian *De plantis*^17^. It was further developed throughout the Middle Ages, culminating in a more concrete and wide-ranging application to the vegetal world in Albert the Great’s *De vegetabilibus*, where a complex and structured doctrine of the so-called *digestio plantarum* was articulated.^18^ The concept of *digestio* made it possible to explain all physiological processes relating to plants, including the formation of each specific organ and part. According to Albert the Great, based on the kind of *digestio* involved—and thus of the kind of moisture and heat involved—it is possible to classify the different categories of plant parts (Panarelli, 2020b). This theoretical framework also informs Dino del Garbo’s account. In his commentary, he identifies the four causes of fruit generation, following the Aristotelian schema of four causes: The material cause of the fruit is a thick and fatty moisture that has been digested and transformed by the plant’s natural heat. The formal cause is the process of digestion (*digestio*), as described by Aristotle in *Meteorologica* IV. The efficient cause is the plant’s own power to digest a consistent, oily or fatty moisture, aided by the celestial heat. The final cause is the preservation of the species through time and by nature, since no individual lives for its own sake, but only insofar as it contributes to the continuation of the species.^19^ More specifically, Dino’s argument then focuses on the two main material and efficient principles (*principia materialia et efficientia*). The material principles include both the seed’s intrinsic moisture and the moisture drawn from the soil. The efficient principle is twofold: internally, the plant develops through an innate heat within the seed (*calor innatus*); externally, it is influenced by the universal heat of celestial bodies and the supportive heat of the earth.^20^ This explanatory model closely aligns with the one elaborated by Albert the Great in his *De vegetabilibus*, in which plant generation is explained through a more articulated set of interacting principles. There, Albert identifies seven such principles: three types of heat (*calor celi*, *calor loci*, and *calor seminis*), three types of moisture (*humor naturalis*, *humor loci*, and *humor pluviarum*), and air (Panarelli, 2020, p. 357). In his explanation, heat functions as the efficient principle, while moisture plays a ministering or instrumental role. Albert also refines the concept of moisture inherent to seeds by using different terms—natural moisture (*humor naturalis*), seminal moisture (*humor seminalis*), and radical moisture (*humor radicalis*) –, depending on the plant organ that is discussed. He distinguishes it from nutrimental moisture, whose functions are fulfilled by the local moisture (*humor loci*) and moisture from rain (*humor pluviarum*). Although this study does not allow for a direct textual dependency to be established between Dino del Garbo’s commentary and Albert’s *De vegetabilibus*, and although Dino’s arguments is less articulated, it does reveal significant conceptual affinities. In both authors, the differentiation of the plant’s parts and functions depends upon the specific kind of heat acting upon a corresponding kind of moisture. In Dino, the essential and generative parts of the plant —such as fruit, seed, and root—derive from an internal heat acting on intrinsic moisture, whereas secondary structures like the bark result from an external and putrefactive heat (*calor putrefactivus*). Albert systematizes this functional distinction by differentiating between *partes integrales essentiales*, ordered to the preservation of the individual plant, *partes essentiales*, directed toward the conservation of the species, and *partes non essentiales*, which serve neither the individual nor the species directly. In both cases, therefore, the hierarchy and function of vegetal parts are determined by the qualitative distinction between the forms of heat and moisture involved. The different principles explain the division between internal generative elements and external protective elements. The hierarchical organization of vegetal parts may also recall analogous distinctions in medieval embryology, such as Avicenna’s division between spermatic and sanguinary *membra*, where distinct generative principles account for the formation of different bodily parts (Van ’t Land, 2012).^21^  

A closer reading of Hippocrates’ words allows Dino to describe the key physiological condition for fruit and seed production in plants. Fructification occurs, Dino explains, when the plant’s watery moisture has been sufficiently dried and the internal channels have widened enough to allow the passage of thick, well-digested nourishment (*nutrimentum grossum et pingue digestum*). In contrast, if this moisture remains in its thin, watery state and the channels are still narrow, the plant is unable to generate fruit or seed. The reason for this is that the generation of fruit and seed depends on a specific form of digestion—what Aristotle has called *pepansis* in *Meteorologica* IV (Aristoteles, 2008, 379b10), where he describes this process as characteristic of fruit-bearing plants.

Such digestion is only possible when the plant’s internal heat is strong enough to act effectively on the moisture. Early in the plant’s development, when the internal moisture is still abundant, watery, and subtle, and the channels are constricted, the innate heat is weakened and suppressed by the excess humidity. In this phase, the plant produces only leaves—structures suited to the aqueous nourishment that can still pass through the narrow pathways.

Over time, however, as this thin moisture is gradually consumed, the heat within the plant gains strength and becomes capable of digesting the remaining denser, more viscous moisture, thereby transforming it into the substance of fruit and seed. This explains why, in the initial stages, the production of fruit is not yet possible: the matter is too raw and the active principle too diminished. Only later, when the matter has become more apt—thicker and more consistent—and the innate heat is reinforced, also aided by external warmth, can the generative process unfold.

According to Dino, this developmental pattern once more reflects a broader analogy with animal generation: animals are likewise incapable of producing seed in the earliest phases of life due to the immaturity of their material and the weakness of their generative heat.

As in previous cases, Dino del Garbo consistently relies on the analogy with animals, which is foundational to his argument. He observes that the delay in seed production during the early stages of a plant’s life is not solely due to previously discussed factors but mirrors a similar process in animals. Just as animals initially require moisture primarily for growth and nourishment rather than reproduction, plants too lack an immediate surplus of pure moisture suitable for seed formation. At this early stage, the excess moisture is generally impure and unfit for generation. Only as growth progresses—and the demand for expansion decreases—this moisture becomes purified and capable of supporting the production of fruit and seed.

### Two questions regarding the ontological status of seeds

The section of Dino del Garbo’s commentary concerning the generation from seed concludes with two questions of a distinctly philosophical nature, emblematic of the scholastic environment in which the text was conceived. The first question concerns a subtle ontological inquiry: whether the seed from which a plant is generated belongs to the essence of the plant. Posing this question means asking whether a plant can still be defined as such in the absence of seeds.

At the heart of this discussion lies a crucial distinction between the individual and the species. Dino explores whether the seed constitutes a *part* of the plant in any essential sense. Of particular interest is Dino’s reference to Averroes’ *De plantis*^22^, whose transmission remains a puzzle, especially given the unresolved question of whether a Latin translation ever circulated.^23^

Dino del Garbo ultimately distinguishes between two senses of “part”: on the one hand, it can mean something that shares in the form of the whole, for instance, flesh or bone in animals; on the other, it can refer to something that contributes functionally to the whole without formally participating in its essence, for instance, hair or nails.^24^ In the first sense, the seed is not a part of the plant, since it does not participate in the plant’s vegetative soul—it is not formally “animated” or an organic body.

Thus, he introduces a further key distinction:

I then say that there is a twofold mode of being in things: one pertains to individual being, the other to specific being. Accordingly, it must be understood whether something is a part contributing to the being of the individual, or rather to the being of the species.^25^

In terms of *individual* being, then, the seed is extrinsic: a plant remains perfect even in the absence of its seed, just as an animal remains complete without its sperm. However, when considered in relation to *specific* being—that is, the perpetuation of the species—the seed plays an essential role. It ensures the continuity of the species through successive individuals, thereby qualifying as a part in this broader, teleological sense.

The second question arises from empirical observation and addresses a fundamental issue: can plants that normally grow from seed also be generated without it?

The problem is framed in teleological terms—if generation without seed were possible, then seed would seem to exist in vain, contradicting the Aristotelian principle that neither nature nor God acts without purpose.

However, observations in certain wild regions show trees appearing spontaneously without any visible seed. Dino responds affirmatively, supporting his view with both sensory evidence and philosophical reasoning. Just as some animals can be generated with or without seed (as Aristotle notes in *Historia animalium*), so too—*a fortiori*—can plants, which are ontologically simpler. This raises a deeper metaphysical question: are plants generated without seed truly of the same species as those produced by seed? Dino acknowledges that this question remains unresolved and sets it aside for the time being.^26^

He then highlights the role of a particular formative heat that directs the universal celestial influence (*quoddam calidum proportionatum commensuratum a caliditate corporum celestium)* toward generating a specific plant. Thus, even spontaneous generation aligns with the natural order, and the seed’s function is preserved: it enables more efficient reproduction and better maintains the continuity of a species.

This issue can also be viewed as being in line with the position expressed by Albertus Magnus in his *De vegetabilibus*^27^, where, although not cited explicitly by Dino, a clear resonance with his argument can be detected. Albertus discusses the generation of perfect plants from putrefaction without seed, emphasizing that plants, due to their homogeneity, can come into being more easily than animals, which require a fully formed seed according to species and form, while also describing the action of celestial influence on the formative heat involved in generation.

## Reproduction through explantation

Building on Hippocrates’ observations, Dino del Garbo offers a detailed explanation of root formation in cutting-propagated plants, highlighting the crucial role of moisture and an animating spirit (*spiritus*) in this process.^28^ According to Dino, when a branch is cut from a tree and planted, the wound facing toward the soil becomes the origin of soft roots. This transformation occurs as the cutting absorbs humidity from the earth and is “inflated” by a vital spirit. The above-ground portion, initially lacking this spirit and not yet swollen, undergoes a conversion in which moisture and spirit move downward, breaking through the tissue to produce tender roots. As these roots mature and draw moisture upward, the upper part of the plant becomes infused with spirit, enabling leaf growth and further development.

In the hands of Dino del Garbo, what begins as a technical reflection on plant grafting turns into a sophisticated argument on metaphysical implications. In this passage, the medical commentary reveals its philosophical ambition by recurring to the Aristotelian terminology of *actus* and *potentia*, in order to distinguish two fundamentally different kinds of plant generation. While reproduction from seed is described as a paradigmatic instance of generation *simpliciter*, involving the passage from pure potentiality to actuality, grafting or cutting does not yield a new substance but merely sustains an already actualized individual:

There is, in a certain sense, a difference in the generative principles of the two forms of reproduction. In the case of generation from seed, the entire plant is generated from a state of pure potentiality and thus this is generation in the proper sense, as it is a process from being in potency to being in act. In contrast, generation through cutting is not the generation of a whole previously non-existent entity, but only of a part. It is not the production of something from pure potency into act, since the branch that is transplanted already exists in actuality and continues to do so without corruption. Even though it has been severed, it still possesses an actual soul and life and is thus actually informed by a soul. Consequently, cutting does not result in a numerically distinct new plant, but rather preserves the same individual. What occurs is only the generation of a part—namely, the roots—through which the plant maintains its existence by drawing in moisture, from which it is nourished and grows. Therefore, this is not generation in the absolute sense, but only of a part, since it is merely an increase in size.^29^

In this way, Dino infuses medical explanations with the conceptual rigor of scholastic metaphysics. The medical question is not only addressed in terms of the plant’s physiological functioning, but previously also in a metaphysical dimension. The fact that the passage is from act to act allows to define reproduction through explantation as more appropriately aligned with the process of growth than with that of generation. The asymmetry between seed-based and transplant-based reproduction culminates in a remarkable physiological and philosophical reflection. Hippocrates, as interpreted by Dino, observes that in plants generated from seed, foliage appears before roots. This phenomenon is explained by the abundant internal moisture already present in the seed—as explained above—which temporarily sustains growth without external nutrition. In contrast, a transplanted branch, already an *ens actu* and not a being arising from potentiality, lacks both this intrinsic moisture and the ontological condition of a beginning. Therefore, it must first establish roots to draw in new nourishment, before any visible vegetative growth can occur.

Furthermore, Dino del Garbo deepens the physiological contrast between plants and animals by highlighting a crucial feature of the vegetal life: the continuous regeneration of parts (*generatio partium continua*). This capacity, he explains, is what enables plants—or even severed parts of them—to survive, grow, and be perfected over time. Unlike animals, whose severed limbs quickly perish due to a lack of nourishment and structural integrity, plants can regenerate roots that allow them to continue absorbing nourishment. Thus, while certain imperfect animals may briefly survive dismemberment due to their simple composition (and proximity to plant nature), only plants have the ongoing regenerative capacity that allows their severed parts to endure meaningfully.

This line of reasoning culminates in in a philosophically charged conclusion, as Dino draws on Aristotle, and in particular on the *De Anima* 413b16-21, to support his argument:

Therefore, the soul in plants is not simply one, as the Philosopher says, but it is one in act and multiple in potency; hence, in each nodal part of the plant there is a soul through which it can live even when severed. And in this, nature has provided for plants the capacity to multiply.^30^  

In other words, the soul in plants is not simply one, but one in act and multiple in potency. Dino del Garbo, echoing Aristotle, yet subtly reinterpreting him, proposes that in each knot (*nodus*) of the plant, the soul is present in such a way that any portion, once severed, retains the potential for life.

## Conclusion

The analysis of Dino del Garbo’s *Recollectiones in Hippocratem De natura foetus* reveals a strikingly original moment in the confluence of medieval medicine and natural philosophy. While drawing explicitly on Hippocratic and Aristotelian traditions, Dino does not merely comment on received authorities: he actively reshapes their conceptual framework by applying the rigour of scholastic reasoning to the domain of plant generation. He is clearly familiar with the intellectual tradition within which he is working, as evidenced by his references to Aristotelian and pseudo-Aristotelian texts, and more implicitly, by the similarities between his position and that of Albert the Great.

Dino’s treatment stands out precisely due to its dual focus: on the one hand, it pays attention to the empirical and anatomical concerns of the medical tradition; on the other, it attends to the philosophical precision connected to Aristotelian and pseudo-Aristotelian natural philosophy, touching also metaphysical issues. His reflections on seed structure, internal sexual polarity, and vegetative soul distribution reveal a systematic attempt to bridge observations in plant science with the ontological and causal schemes of scholastic thought.

Dino del Garbo’s commentary is the earliest and only known Latin engagement with *De natura foetus*, but it is also a rare integration of plant embryology and metaphysics—a botanical physiology that remains firmly grounded in Hippocratic empiricism while extending its reach through sustained philosophical elaboration. By systematically drawing analogies between plant and animal generation, Dino’s commentary illustrates an emerging method of natural inquiry in which direct observation and anatomical study are underpinned by a philosophical framework that provides their theoretical scaffolding.

## Footnotes

1 On Hippocratic embryology, see Jouanna (2008).

2 The translation is transmitted by the edition of *Articella*, printed in Venice in 1487; see Hippocrates, 1487.

3 See the ms. München, Bayerische Staatsbibliothek, Clm 39: “Incipit liber ypocratis de natura pueri translatus de greco in latinum a magistro Bartholomeo de Messana in Curia illustrissima Manfredi regis Sicilie scientie amatoris de mandato suo”. On Bartholomew of Messina see De Leemans 2014. As demonstrated by Giorgianni, the manuscript *Vat. Gr. 276* preserves the text translated by Bartholomeo, which does not include the Hippocratic treatise *De genitura*, often transmitted alongside *De natura foetus* in the manuscript tradition (Giorgianni ﻿2012b).

4 See the ms. Città del Vaticano, Vat. lat. 4464, f. 124r: “Expliciunt recollectiones super libro de natura foetus Hippocratis reportate sub excellentissimo artium doctore et scientiae medicinae Magistro Dino de Florentia per magistrum Julianum de prehuntis Sub anno domini 1310 die 10 mensis octubris, qui sit benedictus”.

5 The Bologna Medical School was to a certain extent characterized by the activities of ‘Taddeo Alderotti and his Pupils’, which is the title of a masterful work by Nancy Siraisi on this topic; see Siraisi, 1981.

6 On this topic, see Baldassarri, 2022 and Baldassarri, 2023, and also Sandford in this fascicle.

7 Dino del Garbo (1518), 71va: “Plantarum duplex est modus generationis, nam quedam generantur ex semine eiecto in terra, quedam autem per ipsarum explantationem”.

8 See Nicolaus Damascenus (1989); Albertus Magnus (1867). On this, see Panarelli (2020).

9 Dino del Garbo (1518), 71vb: According to this line of reasoning, it should be noted that Hippocrates, in these statements, aims to illustrate the mode of plant generation by drawing a parallel between plants that originate from seed and animals that develop inside the womb. “Iuxta quam partem, notandum quod Hyppocrates in his verbis, volens ostendere modum generationis plante, vult ostendere similitudinem plantarum, que oriuntur ex semine, et animalium, que oriuntur in matrice”.

10 Dino del Garbo (1518), 71vb: This analogy first operates with location and then with the dispositions that occur in the process of generation. “Hec autem similitudo est primo quantum ad locum; secundo quantum ad dispositiones, que fiunt in generatione”.

11 In this article, the English terms “semen” and “seed” are used conventionally to distinguish, respectively, animal and plant reproduction. In the Latin sources discussed here, however, the term *semen* (*seminis*) is employed consistently and often interchangeably in both contexts, including in Dino del Garbo and in the Latin translation of *De natura foetus*.

12 Dino del Garbo (1518), 71vb-72ra: “Et has tres dispositiones ponit Hyppocrates in littera, ut patet verumtamen licet conveniant in predictis. Tamen in hoc est differentia inter ipsa, quia in seminibus et plantis non est distincta virtus masculi a virtute femine, sicut in animalibus, sed confunduntur simul in eodem semine, verumtamen non sunt adhuc iste virtutes sic. confuse in semine, quin in semine sit dare unam partem, que obtinet magis virtutem activi principii, aliam autem, que obtinet locum passivi, ita quod principium activum magis viget in una parte et hoc potest appellari masculus similitudinarie, in alia vero parte magis viget principium passivum, quod potest assimilari femine. Sicut apparet in grano frumenti, quia circa cuspidem magis viget principium activum sive virtus activa quam in alia parte; et ideo formice quando volunt sub terra conservare granum, ne oriatur corrodunt cuspidem grani et dimittunt ipsum et tunc amplius non nascitur germen ex eo. Sed in animalibus perfectis generatis per coytum sunt distinctus masculus a femella sive agens et patiens propter perfectionem eorum; quia requiritur in eis plus de humido et magis depurato quam in plantis”.

13 For a more general discussion of the distinction between female and male principles in Albert the Great, see Cerrito (2023). On plant powers and life, see Baldassarri and Blank (2021).

14 Albertus Magnus (1867), 1.1.12. n. 92, 47: And thus, a certain resemblance to the male and female sex is found in the seed of plants, for there is something warmer than the rest, and this is what forms and acts, and something more humid and colder, which is like the female seed that is acted upon and formed. And in this way alone is there a resembling sexual virtue – not true, but imitative of it – in plants. “Et sic. similitudo quaedam masculini sexus et feminini est in semine plantarum, quia ibi est aliquid reliquo calidius, et hoc est formans et operans, et aliquid humidius et frigidius, et hoc est sicut semen femininum operatum et formatum. Et hoc modo solo sexus virtus, non vera, sed longe imitativa eius, est in plantis”.

15 Dino del Garbo (1518), 72rb: “Ex quo apparet, quod generatio stipitis est prior simpliciter et secundum naturam quam frondis et quam generatio radicis et prius perficitur; et licet frons prius appareat quam stipes, non tamen primo perficitur sed tardius et quod radix tardius apparet quam frons et prius etiam perficitur quam frons, quia frondes ille non vere tarde perficiuntur et cadunt et frondes vere renascuntur et oritur germen, quod postea producit vera folia. Quod ergo hic dicit Hyppocrates est verum quantum ad generationem imperfectam et quantum ad generationem secundum apparentiam et de frondibus, que non sunt vere frondes”.

16 Dino del Garbo (1518), 72ra: “Et causa huius est, quia illa particula, que dicitur frons non vera, generatur ex humido subtili aquoso et aereo, radix autem generatur ex parte humidi magis grossa et terrestri, que descendit ad inferiora, modo quia semen habet a principio multam humiditatem aquosam et aeream; ideo ipsa petens exitum, quando est semen tumefactum ex humiditate propria adaucta ex humiditate attracta a terra, et propter ebullitionem calidi naturalis seminis in humidum magis est nata erumpere semen pars magis subtilis et aquosa et aerea, propter quod primo apparet generatio talis frondis”.

17 Nicolaus Damascenus (1989): The parts of plants are, for the most part, porous, because heat draws moisture toward the extremities of the plant, and the matter is dispersed throughout all its parts. What is excessive then flows outward. As in a bath, where heat draws moisture and converts it into vapor which rises and, when in excess, condenses into droplets, so too in both animals and plants do superfluities ascend from the lower to the upper parts and descend from the upper to the lower in accordance with their respective operations. “Partes autem plantarum secundum plurimum sunt rarae, quia calor humorem ad extremitates plantae trahit dispergiturque materia per omnes partes eius, et quod superfluit emanabit. Ut in balneo calor humorem attrahit et in vaporem convertit, qui elevatur, et quando superfluus fuerit vertetur in guttas, similiter quoque in animali et planta superfluitates ascendunt ab inferioribus ad superiora et descendunt a superioribus ad inferiora in actionibus suis”.

18 Albertus Magnus (1867) 4.1.1, n. 7, 215: Digestion occurs where there is moisture as the subject and heat as the agent. And when they reach a proportionate state in accordance with the nature of the thing, digestion is said to have taken place. For then the moisture has been brought to completion in accordance with the being of nature, and the heat is retained in it, as in a subject proportionate to it by nature.“Digestio enim est, ubi est humor sicut subiectum, et calor sicut agens. Et quando terminum proportionalem naturae rei consequuntur, tunc facta est digestio, quoniam tunc completum est ad esse naturae humidum, et calor retinetur in illo, sicut in subiecto sibi secundum naturam proportionali”. I have dealt with this topic in Panarelli 2020a.

19 Dino del Garbo (1518), 73va-b: “Causa materialis fructus est humidum pingue digestum et superatum a calido naturali plante. Causa formalis eius est digestio que dicitur ab Aristotele in IV *Metheora pasis*. Causa efficiens est virtus plante digerens humidum constans et unctuosum vel pingue adiuta a calido celesti. Causa finalis est conservare species tempore et natura, quia nullum individuum vivit sibi ipsi, nisi ut conservetur species”.

20 Dino del Garbo (1518), 73vb: The principles that concur in the generation of plants are twofold, just as they concur in the generation of any natural thing – namely, the material and the efficient causes. The material principles of the plant are twofold, corresponding to the two types of moisture found in the seed, from which the plant is generated: one is the proper and intrinsic moisture of the seed itself, and the other is that which is drawn from the earth. Similarly, the efficient cause of plants is twofold: internal and external. The internal cause is the proper heat contained in the substance of the plant’s seed. The external efficient cause is likewise twofold: one is the universal principle, which is the heat of the sun and other stars, by whose power the internal heat of the seed acts; the other is a secondary and cooperative external cause, which is the heat contained in the bowels of the earth, which assists in the digestion of the plant’s nourishment. Now, the principal agent is the universal agent, namely the celestial body, for it is that which gives life – as is clear from the Philosopher in *De animalibus* XVI – where he affirms that in the generation of animals and plants, certain actions proceed from heat. Hence, heat is said to act absolutely, such as to harden, mix, thicken, rarefy, condense, and so forth. “Principia autem concurrentia ad generationem plantarum sunt duo: sicut concurrunt etiam in generatione cuiuslibet rei naturalis scilicet materialia et efficientia. Principia autem materialia plante sunt duplicia: sicut duplex humiditas reperitur in semine, ex quo planta generatur, nam quedam est humiditas propria seminis et intrinseca sibi, ut illa, que a terra attrahitur. Similiter principium agens plantarum est duplex, scilicet intrinsecum et extrinsecum. Intrinsecum est proprium calidum inclusum in substantia seminis plante. Agens autem extrinsecum est duplex, quia quoddam est principium universale et hoc est caliditas solis et aliarum stellarum, in cuius virtute agit calidum intrinsecum seminis; aliud est principium effectivum extrinsecum secondarium et coadiuvans et hoc est calidum inclusum in visceribus terre, quod adiuvat ad digestionem nutrimenti plantarum. Nunc autem agens principale est agens universale, scilicet corpus celeste, nam id est quod dat vitam, ut patet per philosophum XVI *De animalibus*, ubi vult Philosophus, quod in generatione animalium et plantarum sunt quedam actiones, que proveniunt a calido, unde calidum est absolute, ut indurare, permiscere, ingrossare, subtiliare, condensare etc.”

21 Avicenna (1507, 7ra) discusses this issue in the chapter titled ‘Quid sit membrum et sue partes’. On Avicenna’s explanation of plant functions see Alpina (2021 and, 2022). More in general on medieval embriology see Martorelli Vico (2022).

22 Dino del Garbo (1518), 73vb: The argument in favor relies on Averroes’ *De plantis*, in which he appears to suggest that both the seed and the fruit are to be considered parts of the plant. “Et arguitur, quod sic. auctoritas Averrois in libro De plantis, qui videtur velle quod semen et fructus sint partes plante”. However, as already noted by Siraisi (1981, 183 n. 98), the reading 'Averrois' is not attested in BAV, Vat. lat. 4464, and may therefore be due to an error in the printed edition.

23 Nicolaus Damascenus (1989), 363–371.

24 On this seee Aristotle *De partibus animalium* 645a35 and Johansen (2026).

25 Dino del Garbo (1518), 73vb: “Tunc dico quod duplex est esse in rebus, quoddam est esse individuale, aliud est esse specificum, aut ergo intelligitur quod sit pars faciens ad esse individui aut quantum ad esse specificum”.

26 Dino del Garbo (1518), 74ra: A significant difficulty arises here, namely the important question of whether the plant generated from seed is of the same species as that which is generated without seed and through putrefaction. Opinions on this matter differ considerably. However, since this is a physical question, it shall be set aside for now, although it is clear that the two are at least similar. “Sed in hac questione emergit anxioma, quia oritur questio valde magna, scilicet, utrum planta illa que generatur ex semine sit eiusdem speciei cum illa que generatur sine semine et per putrefactionem et de hoc sunt opiniones diverse, tamen quia hec est questio physica ad presens dimittatur, sed saltim videmus quod sunt similes”,

27 Albertus Magnus (1867) 4.3.1, n. 88–92, 255–257. On this, see Panarelli, 2022.

28 On the notion of *spiritus* see Meroni, 2024.

29 Dino del Garbo (1518), 74va: “Et quodammodo differunt quantum ad principia generationis: quantum ad generationem quidem, quia generatio plante ex semine est generatio totius non preexistentis nisi in pura potentia. Et ideo generatio eius est generatio simpliciter, quia est processus ab esse in pura potentia ad esse actu; sed generatio per transplantationem non est alicuius totius non preexistentis, sed solum est generatio partis. Non enim est productio ex ente in pura potentia ad ens actu, quia ramus quando transplantatur est ens actu et manens actu non corruptus. Nam dato quod ramus sit abscisus, habet tamen animam et vitam quam vivit actu, et qua anima informatur actu. Et ideo ex transplantatione non generatur alia planta numero, sed illa eadem numero manet. Sed talis est solum generatio partis, per quam salvatur in esse, quia generantur in ea radices per quas attrahitur humidum, ex quo ipsa nutritur et augetur. Et ideo ibi non est generatio simpliciter, sed partis, quia augetur solum”.

30 Dino del Garbo (1518), 74rb: Ideo anima in ipsis non est simpliciter una, ut dicit philosophus, sed est una in actu plura autem in potentia, unde in qualibet parte nodosa est anima in planta per quam posset vivere decisa et in hoc previdit natura in plantis, ut possent multiplicari. On this see Aristotle (2016), 413b16-21:  For just as in the case of plants, some, when divided, evidently go on living even when separated from one another, there being one soul in actuality in each plant, but many in potentiality, so we see this occurring in other characteristics of the soul in the case of insects cut into two.   
